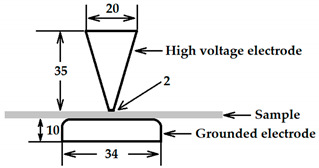# Electrical Property of Polypropylene Films Subjected to Different Temperatures and DC Electric Fields

**DOI:** 10.3390/polym13172956

**Published:** 2021-08-31

**Authors:** Chuyan Zhang, Weichen Shi, Qiao Wang, Mingguang Diao, Huseyin R. Hiziroglu

**Affiliations:** 1 School of Information Engineering, China University of Geosciences (Beijing), Beijing 100083, China; 1005171131@cugb.edu.cn (W.S.); 1001171225@cugb.edu.cn (Q.W.); dmg@cugb.edu.cn (M.D.); 2 Department of Electrical & Computer Engineering, Kettering University, Flint, MI 48504, USA; hhizirog@kettering.edu

**Keywords:** polypropylene film, DC electric field, temperature, breakdown, aging, electrical insulation

## Abstract

A polypropylene (PP) film is usually used as a dielectric material in capacitors as well as cables. However, PP films may degrade because of the combined effect of temperature and electric field. In an earlier study, plain PP films and PP films loaded with nano-metric natural clay were studied under sinusoidal (AC) electric fields at power frequency and temperatures above the ambient. To better understand the electrical characteristics of PP film under various conditions, the objective of this study is to determine the time-to-breakdown of the plain PP and PP filled with 2% (wt) natural nano-clay when subjected to time-invariant (DC) electric fields at elevated temperatures. In order to achieve this objective, the effects of uniform as well as non-uniform electric fields were compared at the same temperature for the PP film. In this study, experimental results indicated that the time-to-breakdown of all PP films, plain or filled with nano-clay, decreases with the increase in electric field intensity, non-uniformity of the electric field, and temperature. It was also found that the time-to-breakdown of PP film filled with 2% (wt) natural nano-clay under DC electric field is longer and less sensitive to temperature. Furthermore, when compared with the results under the uniform electric field, PP film filled with 2% (wt) nano-metric natural clay indicates shorter time-to-failure under non-uniform DC electric fields. Finally, the morphology of the samples was observed by digital camera, optical micrography, and SEM, to better understand the mechanism of the breakdown.

## 1. Introduction

For its economic development, the ever-increasing demand for electrical energy in China requires the upgrading of electrical energy infrastructure, especially with the incorporation of renewables sources such as wind and solar energy. A high-voltage direct current (HVDC) transmission systems is one of the most important candidates to reliably deliver large amounts of energy over thousands of miles [1]. When electrical energy is brought to the vicinity of a highly populated area, the transmission is done usually with cables. In this context, HVDC cables are widely used in power systems. These cables require insulating materials having the capability to support the weight of the conductor at elevated temperatures with excellent dielectric properties [2].

Polymeric materials are often used as insulation materials in cables in high-voltage applications. As one of the most widely used polyethylenes (PE) [3,4,5], the aging and electrical properties of cross-linked polyethylene (XLPE) are of great concern [6,7,8,9]. Compared to PE, PP has a relatively high melting temperature that might possibly facilitate higher current-carrying capacity in addition to other micro-structure properties, such as crystallinity, amorphous phase, as well as crystalline/amorphous interface [10]. Moreover, it is known that the overall properties of the polymer seem to be enhanced significantly by the modification of nano-particles [11,12,13]. Thus, among a wide variety of industrial applications, PP film has found a place in electrical applications such as radio-frequency (RF) capacitors [14,15] and power cables due to its seemingly good polarization of electric charge when subject to electric fields. However, RF capacitors and power cables possibly operate at relatively high temperatures above the ambient temperature. It is a well-known fact that through degradation mechanisms, the properties of materials suffer and irreversible changes may take place. Thus, PP films may degrade because of the combined effect of temperature and electric field [16,17].

In an earlier study, plain PP films and PP films loaded with nano-metric natural clay were studied under sinusoidal (AC) electric fields at power frequency and temperatures above the ambient. However, to the best of our knowledge, there is very little information about the behavior of PP and its nano-composites with natural clay under time-invariant electric fields (DC). It has been shown that in solid dielectrics, the development of charge and the breakdown phenomenon are significantly different under time-invariant (DC) electric fields than under sinusoidal electric fields.

High temperature may lead to the degradation of electrical insulation [18,19]. Especially, in the presence of an electric field, this degradation may cause the reduction of the life of the material, which is known as time-to-breakdown or time-to-failure. The prediction of time-to-breakdown or time-to-failure of an insulating material is important since, in practice, one can determine as to when the insulating material is going to fail. Thus, in power systems, in order to avoid unnecessary service interruption, a proper maintenance program can be established to replace the aged material before it fails. Our previous studies have shown that the electrical properties of PP films have been significantly improved by mixing with 2 wt % natural nano-clay [20]. However, a reduced lifespan of nano-composite has been found under combined electric and temperature field, compared to the unfilled isotactic PP film [21]. Consequently, more evidence at more experimental conditions is needed to verify the above conclusion. 

In this work, the same samples were tested under combined DC fields and thermal stresses. Experiments were carried out under different degrees of homogeneity of the electric field. Furthermore, the mechanism of insulation failure can be fully understood by observing the morphology of the experimental samples. The objectives of this study are to have a more comprehensive understanding of various phenomena and electrical properties in polymer-based materials when subjected to DC electric fields, and to lay a solid foundation of these materials before they are applied to real applications in power equipment.

## 2. Materials and Methods

### 2.1. Material Preparations

Preparation of the PP film and its nano-composites tested in this work are the same as in our previous studies [20,21]. The plain isotactic PP films (Pro-fax HL-451H from Basell) were labeled as one group, and 2 wt % natural nano-clay (montmorillonite or known as Cloisite^®^20A, [Al_1.67_ Mg_0.33_ (Na_0.33_)]Si_4_ O_10_ (OH)_2_) filled PP films were labeled as the other group. The dimension of samples was 50 mm × 50 mm with a thickness of 135 μm with a variation of ±10%.

### 2.2. Methods

**Figure 1:**
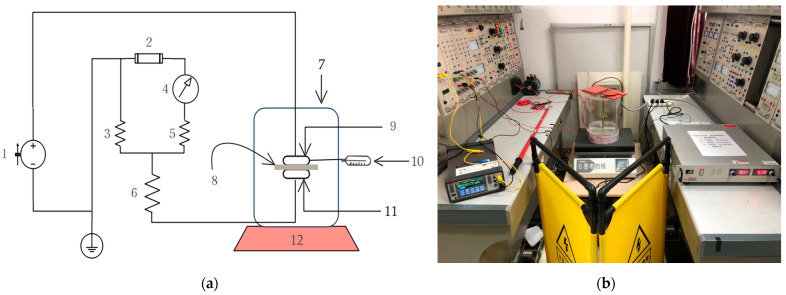
Experimental setups. (**a**) Schematic diagram. (1) high voltage direct current power supply; (2) fuse; (3) divider resistor; (4) digital multimeter; (5) divider resistor; (6) divider resistor; (7) experimental tank; (8) test sample; (9) high voltage electrode; (10) thermocouple; (11) grounded electrode; (12) hotplate; (**b**) photo of the experimental setup.

Figure 1 provides the schematic diagram and photograph of the experimental systems for the failure tests under combined electric field and thermal stresses. A high voltage DC power-supply (DL10∗1200, Wisman, Xi’an, China) with a rated output of 10 kV and a ripple coefficient of less than 0.1% was used to produce electric fields. The applied voltage to the sample was measured by the module inside the power supply and the accuracy of the measurement was better than 0.1%.

In the specially made experimental tank, as shown in Figure 1, a PP film was placed between the upper electrode and the lower electrode. The experimental tank was made of polymethyl methacrylate with an inner diameter of 260 mm, an inner height of 260 mm, and a thickness of 1 mm. The main purpose of using the tank is to keep the environment around the sample relatively stable, so as to reduce the impact of the surrounding environment of the laboratory.

Two groups of stainless-steel electrodes were used in the experiment to compare the results under different electric field conditions. The parameters and side view of the electrodes during the measurement are given in detail in Table 1. Each electrode of the first group was generally in the shape of a circular plate with curved edges so that the fringing effect was minimized to ensure almost a uniform electric field distribution in the test sample to avoid corona discharges. The grounded electrode of the second group was the same as of the first group, while the high voltage electrode of this group had a conical shape capped with a 2 mm diameter circular plate. 

As shown in Figure 1, the experimental tank was placed on a hotplate (DB-2EFS, Lichen, Shaoxing, China) and the hotplate was set to the desired temperature. The temperature was raised from ambient temperature until it was stable and almost constant at approximately 58 °C, 72 °C, and 92 °C on the electrodes. A thermocouple (KT300, Zhongshan Hyperda Technology Co., Ltd., Zhongshan, China) was used to measure the temperatures on the surface of the electrodes through an adaptor in the cylinder wall of the experimental tank. The conduction of temperature is a relatively slow process. In the arrangement shown in Figure 1, the temperature on the electrodes was measured continuously without applying voltage. When the temperature of the electrode reaches the expected value, the continuous rise of the electrode temperature can be controlled by effectively adjusting the output of the hotplate. By this method, the temperature variation on the electrodes can be within 2 °C within 48 h.

During each test, when the temperature was stable at the pre-set level, the sample was subjected to DC electric fields of 40 kV/mm, 50 kV/mm, and 60 kV/mm, respectively. The leakage current was measured by a digital multimeter (DMM6500, Keithley, A Tektronix Company, Cleveland, Ohio, OH, USA) with an accuracy of better than 0.25%.

The time-to-breakdown, which means that the time from the beginning of voltage application to the breakdown (failure) of the sample, was recorded at each temperature and electric field. Five samples were used for each test. 

## 3. Results and Discussion

### 3.1. Time-to-Breakdown of PP Films under Combined DC and Temperature Field

#### 3.1.1. Variation of Time-to-Breakdown with DC Field Intensity at Fixed Temperature

**Figure 2:**
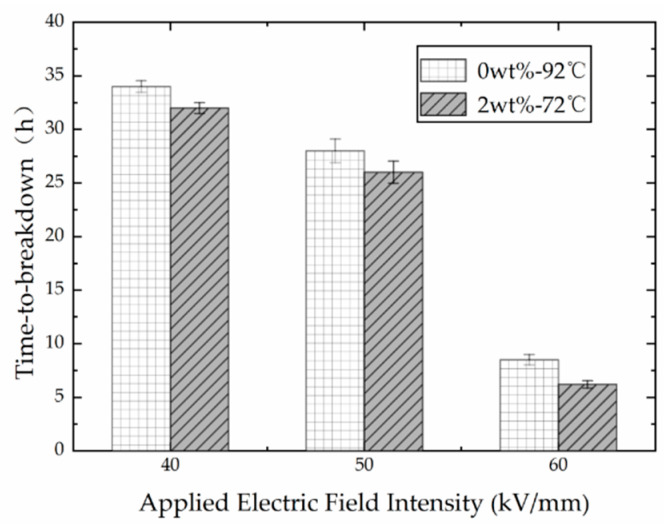
Time-to-breakdown vs applied electric field intensity of the compared two groups.

The time-to-breakdown of both unfilled isotactic PP films and nano-modified PP films decreased with the increase in electric field intensity at a fixed temperature; the results can be found in Figure 2. The experimental results of the two groups showed a similar trend. Due to the limited number of samples, the experiments of the unfilled PP film and its nano-composite were carried out at 92 °C and 72 °C, respectively. When the electric field intensity increased to 60 kV/mm, the time-to-breakdown of PP film decreased to less than 10 h.

It is worth noting that, at the same electric field intensity, the time-to-breakdown of unfilled PP film at 92 °C was longer than that of the nano-modified PP film at 72 °C. This phenomenon is consistent with the discovery under AC field [21], and can be explained by the reason that the coupling between nano fillers and polymer matrix is not strong enough, which leads to the dissociation of nano fillers under long-term electric field and high temperature. Subsequently, the nanoparticles separated from the matrix and aggregated, which led to the weak point of the material.

#### 3.1.2. Variation of Time-to-Breakdown with Temperature at Fixed DC Field Intensity

**Figure 3:**
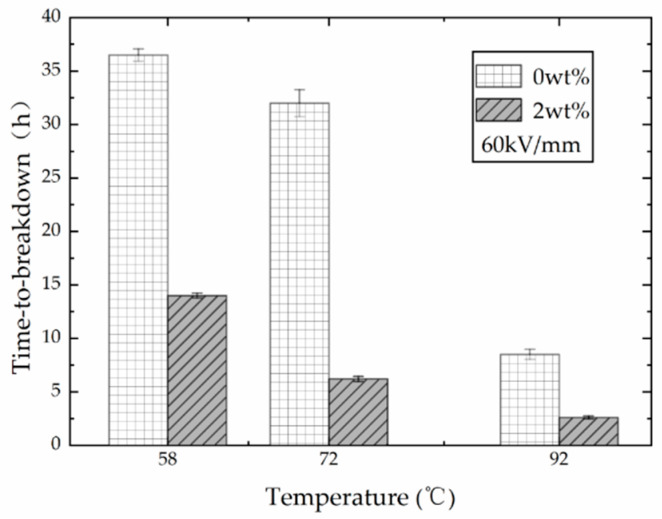
Time-to-breakdown vs temperature at a fixed electric field intensity.

At 60 kV/mm, the time-to-breakdown of PP film and its nano-composite at three different temperatures is shown in Figure 3. The time-to-breakdown of samples mixed with 2 wt % nano-clay particles was shorter than that of the unfilled PP film. The differences in the values of time-to-breakdown between the two groups were 22.5 h, 25.8 h, and 5.9 h, respectively. Consequently, the lifespan of the PP film was shortened after nano-modification under DC voltage, which is similar to the results obtained at AC voltage. Moreover, with the increase in temperature, the time-to-breakdown decreased for both experimental groups. The lifespan of the unfilled samples was more sensitive to temperature, and it decreased more with the increase in temperature. When the temperature reached above 90 °C, the time-to-breakdown of both groups was less than 10 h under DC voltage.

Due to the symmetrical molecular structure, the crystallinity of isotactic PP is higher than that of non-isotactic pp. In the filled PP films, the presence of nanoparticles has a negative effect on the crystallinity of PP. Moreover, the temperature field provides external energy, which increases the molecular thermal motion. The polymer matrix and nano-clay filler have different motion activity, making the filled PP more unstable than the plain PP at the same temperature. As a result, the lifespan of PP films filled with nano-clay, which has a higher molecular mobility, is shorter than that of the plain samples under combined electric and temperature fields. 

### 3.2. Comparision of the Results between AC and DC Electric Fields

#### 3.2.1. Time-to-Breakdown

**Figure 4:**
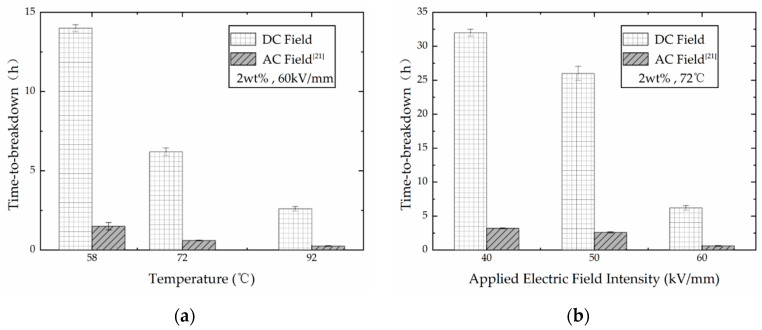
Results of PP film filled with 2 wt % nano-clay under DC and AC electric fields (data under AC electric fields are from [21]). (**a**) Results at 60 kV/mm; (**b**) results at 72 °C.

Under different electric fields, dielectric polarization has different performance. The comparison of the experimental results of PP films filled with 2 wt % nano-clay under DC electric field and AC electric field is shown in Figure 4.

The aging of the polymer was greatly accelerated by high temperature and high electric field intensity. It can be seen from Figure 4 that the time-to-breakdown of the PP film filled with nano-particles under DC voltage was much longer than that under AC voltage. At a fixed electric field intensity, the time-to-breakdown of the nano-modified samples under DC electric field was approximately 10 times more at three different temperatures compared with the results measured under the AC electric field. Simultaneously, the time-to-failure of nano-modified samples under the DC electric field at three different electric field intensities was also approximately 10 times more compared with the results under the AC electric field at a fixed temperature.

The above results indicated that the PP film is more prone to failure under the AC field. This could possibly be because of the internal energy loss due to the rate of the charge of polarization processes in the dielectric under AC electric fields. When the material is subjected to DC voltage, only the leakage conduction loss dominated the internal energy loss of the dielectric. However, under AC voltage, there is polarization loss in addition to the leakage conduction loss. More energy loss of the polymer under AC field accelerates the temperature elevation inside the material, which further intensifies the thermal motion of the molecules. Admittedly, the nano-clay fillers in the samples have different physical and chemical properties from the polymer matrix. Subsequently, the enhanced thermal motion of all molecules in the PP film will destroy the diffused ionic layer between the clay flakes and the polymer matrix. As a result, a large number of clay flakes peel off from the polymer matrix, which accumulate and collide with the polymer molecules. Therefore, the weak point is more likely to be produced in the PP film, which will lead to the breakdown under AC voltage.

In addition, due to the different polarization performances under different electric fields, the PP film subjected to time-invariant (DC) electric fields has lower molecular mobility than that of the PP film subjected to sinusoidal (AC) electric fields.

#### 3.2.2. Breakdown Mechanism

**Figure 5:**
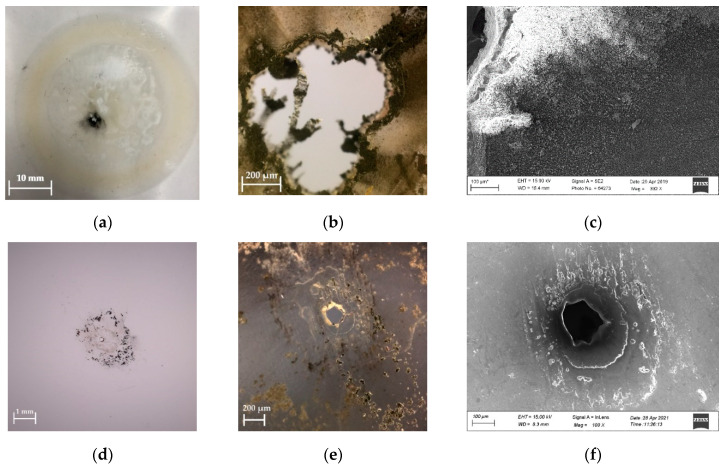
The surface morphology of the breakdown-point for samples after being tested under DC and AC electric field. (**a**) Ordinary photo obtained by digital camera of samples after being tested under AC electric field [21]; (**b**) magnification photo obtained by optical microscope of samples after being tested under AC electric field [21]; (**c**) SEM image of samples after being tested under AC electric field; (**d**) ordinary photo obtained by digital camera of samples after being tested under DC electric field; (**e**) magnification photo obtained by optical microscope of samples after being tested under DC electric field; (**f**) SEM image of samples after being tested under DC electric field.

The surface morphology of the breakdown-point obtained by digital camera, optical microscope, and scanning electron microscope (SEM) of PP film filled with 2 wt % nano-clay under the combined electric and temperature field is shown in Figure 5. 

The failure of polymers in the long-term combined electric and temperature field is a time-dependent phenomenon as well as a process of thermal breakdown. The temperature field provided further energy for the internal energy loss and accelerates the aging of materials. Compared to the breakdown-point of samples after being tested under DC electric field, it is indicated that the breakdown-point of samples after being tested under AC electric field has bigger size, shown in Figure 5. Moreover, more serious carbonization of the surrounding material of the breakdown-point of samples after being tested under AC electric field can be found. These results were attributed to the more internal energy loss of the samples under AC electric field. 

Figure 5 also shows that the color of the PP film changes from transparent to light yellow in the contact area of the electrode after the AC experiment, while the color of the sample in this area does not change significantly after the DC experiment. Therefore, the aging of materials under DC voltage is less sensitive to temperature compared with that of the test under AC voltage.

### 3.3. Comparision of the Results between Uniform and Non-Uniform Electric Fields

#### 3.3.1. Time-to-Breakdown under Different Electric Field Uniformity

**Figure 6:**
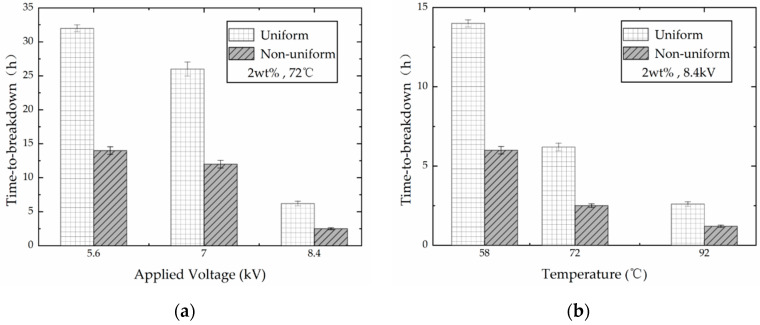
Time-to-breakdown of PP films filled with 2 wt % nano-clay between uniform and non-uniform electric field under DC voltages. (**a**) Results at 72 °C; (**b**) results at 8.4 kV.

Based on the comparison of the results under different electric field formations, it is found that the nano-modified PP film has a longer lifespan under DC electric field at a fixed experimental condition. This provides more possibilities for the application of PP in DC power equipment. However, the distribution of the electric field in the insulating material under actual operating conditions is extremely complex, and the non-uniform electric field distribution is often encountered. Consequently, in order to fully understand the characteristics of PP under DC electric field, experiments under different electric field uniformity were carried out. The results are shown in Figure 6.

Experimental results showed that the time-to-breakdown of nano-modified PP film shortens with the increase of the non-uniform DC electric field intensity. Similarly, when the applied voltage is constant, the time-to-breakdown shortens with the increase of the temperature. Therefore, the breakdown characteristics of the material under the non-uniform electric field are also similar to those under the uniform electric field. Experimental results in Figure 6 shows that the time-to-breakdown of the samples under the non-uniform electric field is approximately half of the results under the uniform electric field. However, the value is still greater than the results under the uniform AC electric field.

In the uniform electric field, the breakdown of the sample may occur at any position between the electrodes. However, when the non-uniform electric field was applied, the breakdown always occurs at the tip of the conical electrode where the electric field intensity is much higher than other locations. Consequently, in practical application, improving the electric field distribution on the polymers is an effective way to extend the lifespan of the insulation system.

#### 3.3.2. Discussion

**Figure 7:**
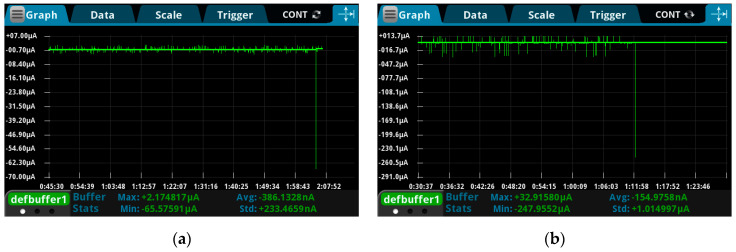
The maximum leakage current before breakdown under DC electric field. (**a**) The value under the uniform electric field; (**b**) the value under the non-uniform electric field.

The maximum leakage current before breakdown was recorded in the DC experiment, and it is found that the value of the leakage current under the non-uniform electric field is 248.0 μA which is approximately 4.5 times of that under uniform electric field (65.6 μA), as can be seen in Figure 7.

Under a long-term DC electric field, the current through the experimental circuit is mainly the leakage current through the sample. No polarization current can be measured. Before the failure of the material, the amplitude of leakage current is extremely small and is even lower than the magnitude of the noise. As the activation energy of the molecules in the material continuously accumulates at an elevated temperature, the physical and chemical properties of polymers are also changing. When the activation energy exceeds the initial barrier of electrical destruction, the breakdown occurs. This process takes more time in DC electric field than in AC electric field. Because in the AC electric field, the increase of activation energy of polymer molecules is not only supported by the temperature field, but also assisted by the polarization current.

**Figure 8:**
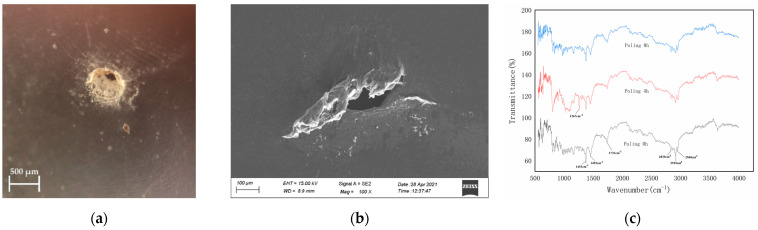
The surface morphology of the breakdown-point for sample after being tested under non-uniform DC electric field and the assessed results of the FTIR. (**a**) The optical microscope photo; (**b**) the SEM image; (**c**) results of the FTIR.

Different from the experimental results obtained under the uniform electric field as shown in Figure 5e,f, the carbonization phenomenon around the breakdown-point of nano-modified PP film is not obvious, as shown in Figure 8a,b. Subsequently, the breakdown of the material is more like the electrical breakdown due to the electric field than the thermal breakdown, although the elevated temperature still has some contribution to the breakdown. Consequently, the breakdown of materials is the result of the joint contribution of electric field and temperature field in uniform electric field. However, the electric field contributes more to the breakdown of materials in the non-uniform electric field. 

The results of Fourier Transform Infra-Red (FTIR) (IRTracer-100, Shimadzu, Japan) shown in Figure 8c can reveal whether the phase state of the internal structure of the material has changed during the experiment. With the increase of polarization time, some bonds in polymers may be deformed and vibrated asymmetrically and causing the PP macromolecular chain to break into some small molecular chains during the experiment, however, the content of most characteristic peaks does not change significantly, which indicated that the structural properties of most C–H bonds and C=O bonds can maintain stability without phase structure changing. The infrared spectrum curves of the three samples have highly consistency in the peak position, the wave number, and the peak shape. As a result, there is no phase structure changing during the polarization process under the non-uniform field. Therefore, the time-to-breakdown of the nano-modified polymer film under the non-uniform DC electric field is less sensitive to the temperature.

According to the kinetic theory of solid strength combined with nonlinear stress-strain dependence [22], the PP film has an accelerated aging process at elevated temperature compared with the results at room temperature. As evident from FTIR spectroscopy, the broken bonds due to the electric field and elevated temperature essentially change the morphology of the material, as shown in micrographies, leading to its rapid aging and to its ultimate breakdown. 

## 4. Conclusions

In this paper, the electrical properties of the PP films under DC electric field combined with temperature field are investigated experimentally. One of the important conclusions of this work is that the nano-modified PP film under DC electric field has a longer lifespan compared to the values measured under AC electric field. The main reason for this result is that besides the leakage conduction loss, under AC electric field, the polarization loss plays an important role in the failure process. Whereas, under DC electric field, only the leakage conduction loss dominates the failure process.

Similar to the experimental results under AC electric field, the time-to-breakdown of the PP film filled with nano-particles is shorter than that of the unfilled isotactic sample. The failure of polymers in the long-term combined electric and temperature field is a time-dependent phenomenon, in which thermal effects contribute to the breakdown. The temperature field provides further energy for the internal energy loss and accelerates the aging of materials. The carbonization at the breakdown point can be used as one of the characteristics to judge the breakdown type of polymers. When a thermal breakdown occurs, there is obvious carbonization at the breakdown point, but when an electric breakdown occurs, it is difficult to observe the trace of carbonization.

Compared with the results under the uniform electric field, the life of the nanocomposite film under the non-uniform DC electric field is shorter. The breakdown of materials is the result of the joint contribution of both the electric field and the temperature field in a uniform electric field. However, the electric field contributes more to the breakdown of materials in the non-uniform electric field.

The experimental conclusions provided in this paper are expected to contribute to a better understanding of the electrical properties of polymers and their wider application in HVDC power transmission lines.

## Figures and Tables

**Table 1:** Parameters of the electrodes.

Electrode Types	Dimension (mm)
Circular plate-to-circular plate	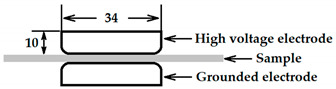
Cone-to-circular plate